# The Semen Microbiome and Its Relationship with Local Immunology and Viral Load in HIV Infection

**DOI:** 10.1371/journal.ppat.1004262

**Published:** 2014-07-24

**Authors:** Cindy M. Liu, Brendan J. W. Osborne, Bruce A. Hungate, Kamnoosh Shahabi, Sanja Huibner, Richard Lester, Michael G. Dwan, Colin Kovacs, Tania L. Contente-Cuomo, Erika Benko, Maliha Aziz, Lance B. Price, Rupert Kaul

**Affiliations:** 1 Department of Pathology, Johns Hopkins School of Medicine, Baltimore, Maryland, United States of America; 2 Center for Microbial Genetics and Genomics, Northern Arizona University, Flagstaff, Arizona, United States of America; 3 Center for Microbiomics and Human Health, Translational Genomics Research Institute, Flagstaff, Arizona, United States of America; 4 Department of Medicine, University of Toronto, Toronto, Canada; 5 Department of Biology, Northern Arizona University, Flagstaff, Arizona, United States of America; 6 Maple Leaf Medical Centre, Toronto, Canada; 7 Department of Environmental and Occupational Health, George Washington University School of Public Health, Washington, D.C., United States of America; 8 Department of Medicine, University Health Network, Toronto, Canada; Vaccine Research Center, United States of America

## Abstract

Semen is a major vector for HIV transmission, but the semen HIV RNA viral load (VL) only correlates moderately with the blood VL. Viral shedding can be enhanced by genital infections and associated inflammation, but it can also occur in the absence of classical pathogens. Thus, we hypothesized that a dysregulated semen microbiome correlates with local HIV shedding. We analyzed semen samples from 49 men who have sex with men (MSM), including 22 HIV-uninfected and 27 HIV-infected men, at baseline and after starting antiretroviral therapy (ART) using 16S rRNA gene-based pyrosequencing and quantitative PCR. We studied the relationship of semen bacteria with HIV infection, semen cytokine levels, and semen VL by linear regression, non-metric multidimensional scaling, and goodness-of-fit test. *Streptococcus*, *Corynebacterium*, and *Staphylococcus* were common semen bacteria, irrespective of HIV status. While *Ureaplasma* was the more abundant Mollicutes in HIV-uninfected men, *Mycoplasma* dominated after HIV infection. HIV infection was associated with decreased semen microbiome diversity and richness, which were restored after six months of ART. In HIV-infected men, semen bacterial load correlated with seven pro-inflammatory semen cytokines, including IL-6 (*p* = 0.024), TNF-α (*p* = 0.009), and IL-1b (*p* = 0.002). IL-1b in particular was associated with semen VL (*r^2^* = 0.18, *p* = 0.02). Semen bacterial load was also directly linked to the semen HIV VL *(r^2^* = 0.15, *p* = 0.02). HIV infection reshapes the relationship between semen bacteria and pro-inflammatory cytokines, and both are linked to semen VL, which supports a role of the semen microbiome in HIV sexual transmission.

## Author Summary

The classical paradigm of HIV infectivity centers on the blood HIV RNA viral load. However, while other fluid compartments such as semen and cerebrospinal fluid can have distinct viral loads from blood, the causes of localized HIV shedding are not fully understood. Since the semen viral load is an independent predictor of HIV transmission risk, it is critical to understand the local factors that trigger increased semen viral shedding in order to develop novel preventative strategies. Here, we evaluated the semen microbiome, bacterial load, and cytokine levels in 22 HIV-uninfected men who have sex with men (MSM) and in 27 HIV-infected MSM before and after initiation of antiretroviral therapy (ART). We found that HIV infection reduces semen microbiome biodiversity, which is restored with ART and immune reconstitution. We also found that semen bacterial load in untreated, HIV-infected men is associated with the levels of seven semen cytokines, relationships not seen in the uninfected controls. In particular, the cytokine IL-1b was uniquely correlated with both semen bacterial and viral load. Our findings support the interaction between semen microbiome and local immunology, and suggest that IL-1b could be a mechanism for semen microbiome to trigger semen viral shedding.

## Introduction

Semen is an important vector in the sexual transmission of HIV [1], and the risk of transmission increases with the semen HIV RNA viral load (VL) [2], [3]. The semen VL is more variable over time than that of blood, and the two are only moderately correlated [4]–[8]. Therefore, since the semen HIV VL is an independent predictor of HIV transmission risk [3], a better understanding of the local factors that trigger or increase HIV shedding in the semen could enable novel interventions to reduce HIV sexual transmission.

Many viral and bacterial pathogens are known to increase HIV shedding in men. Specifically, local reactivation and replication in the genital tract of persistent herpes viruses, such as Cytomegalovirus, Epstein-Barr virus, and Herpes Simplex virus types 1 and 2 [9]–[11], as well as infection by classical sexually transmitted bacterial pathogens, such as *Chlamydia trachomatis* and *Neisseria gonorrhoeae* [12] have been associated with increased semen VL in untreated men. These pathogens are thought to act directly through interaction with HIV-infected CD4+ T-cells [13], [14] or indirectly by local immune activation and recruitment of HIV susceptible cells to the genital mucosa [9]–[12].

However, in HIV-infected men who have sex with men (MSM) on suppressive antiretroviral therapy (ART), T cell activation in the semen has been associated with transient bursts of semen viral replication, in the absence of classical sexually transmitted infections and independent of cytomegalovirus reactivation or herpes infection [6]. There is also evidence that increased proinflammatory cytokine and chemokine in the semen might enhance local HIV replication and evolution in the male genital tract [15]. Together, these findings highlight that compartmentalized factors within the male genital tract could cause immune activation in the semen and are responsible for subsequent increases in HIV shedding.

In addition to spermatozoa, the semen contains nutrients, numerous immune factors, and communities of bacteria [16]–[18]. Studies of infertility have shown a wide range of bacteria in the semen [16], [17], [19], including those hypothesized to cause inflammatory obstructive processes in the male genital tract, such as *Chlamydia*, *Ureaplasma*, and *Mycoplasma* [20], [21]. The semen microbiome in heterosexual men exhibits high prevalence and abundance of commensals, such as *Ralstonia*, *Anaerococcus* and *Corynebacterium*, as well as bacteria abundant in the vagina, such as *Prevotella* and *Lactobacillus* [19].

This study aimed to determine how HIV infection and suppressive ART impact the semen microbiome, and whether the semen microbiome might be associated with inflammation and the VL in semen. We hypothesized that the semen bacterial microbiome represents an important cause of local immune activation, and that this might contribute to the high degree of variability in semen HIV levels. To test this hypothesis, we compared the semen bacteria in 22 HIV-uninfected MSM controls versus 27 HIV-infected, treatment-naïve MSM, and we further examined the semen bacteria in the latter group at one and six months after antiretroviral therapy initiation.

## Results

### Study participants

We enrolled 27 HIV-infected, ART-naïve men and 22 HIV-uninfected control men. All participants were men who have sex with other men (MSM) and the ages of HIV-infected and uninfected men were similar (*HIV-infected Mean* = 36.4 years; *HIV-uninfected Mean* = 34.0 years, *p* = 0.399). Among the HIV-infected men, the baseline seroprevalences of HSV-2 and CMV were 48.3% and 79.3%, respectively. The baseline CD4^+^ T-cell count was 300 cells/mm^3^ (*Range* = 60–610), with median HIV-1 RNA loads of 30,122 copies/ml in blood (*Range* = 435–500,000) and 3,192 copies/ml in semen (*Range* = <300–208,152) (**Table 1**).

**Table 1:** Clinical parameters of the HIV-positive participants at baseline, prior to initiation of antiretroviral therapy.

	ART-naïve men (n = 27)
	*n (%)*
**Age**	
21–29	3 (11.1)
30–39	17 (63.0)
40–49	6 (22.2)
≥50	1 (3.7)
**HSV-2 co-infection**	
Yes	13 (48.1)
No	14 (51.9)
**CMV co-infection**	
Yes	24 (88.9)
No	1 (3.7)
Unknown	2 (7.4)
**Absolute CD4+ T-cell count (/mm^3^)**	
<200	4 (14.8)
200–299	9 (33.3)
300–399	6 (22.2)
400–499	5 (18.5)
≥500	3 (11.1)
**CD4:CD8 T-cell Ratio**	
<0.25	6 (22.2)
0.25–0.50	14 (51.9)
>0.50	6 (22.2)
Unknown	1 (3.7)
**Blood viral load (log10 RNA copies/mL)**	
<4	8 (29.6)
4–4.9	11 (40.7)
≥5	8 (29.6)
**Semen viral load (RNA copies/mL)**	
<4	19 (70.4)
4–4.9	6 (22.2)
≥5	2 (7.4)

Prior to ART initiation, all 27 HIV-infected participants had detectable HIV-1 RNA in the blood and semen. In addition, the blood VL correlated positively with the semen VL (*r^2^* = 0.27, *p* = 0.003); however, while the blood CD4+ T-cell count correlated negatively with the blood VL (*r^2^* = 0.27, *p* = 0.003), it showed no significant association with semen VL (**Figure S1**). The clinical response to ART was excellent. After one month of treatment, viral RNA was only detectable in the semen of seven men and in the blood of 15 men, which further decreased to only one and two men, respectively, after six months of ART. Concurrently, the blood CD4^+^ T-cell count increased to a median of 360 cells/mm^3^ after one month of ART (*Range* = 100–910) and to 450 cells/mm^3^ after six months of ART (*Range* = 275–940).

### Semen bacteria in HIV-uninfected participants

**Figure 1:**
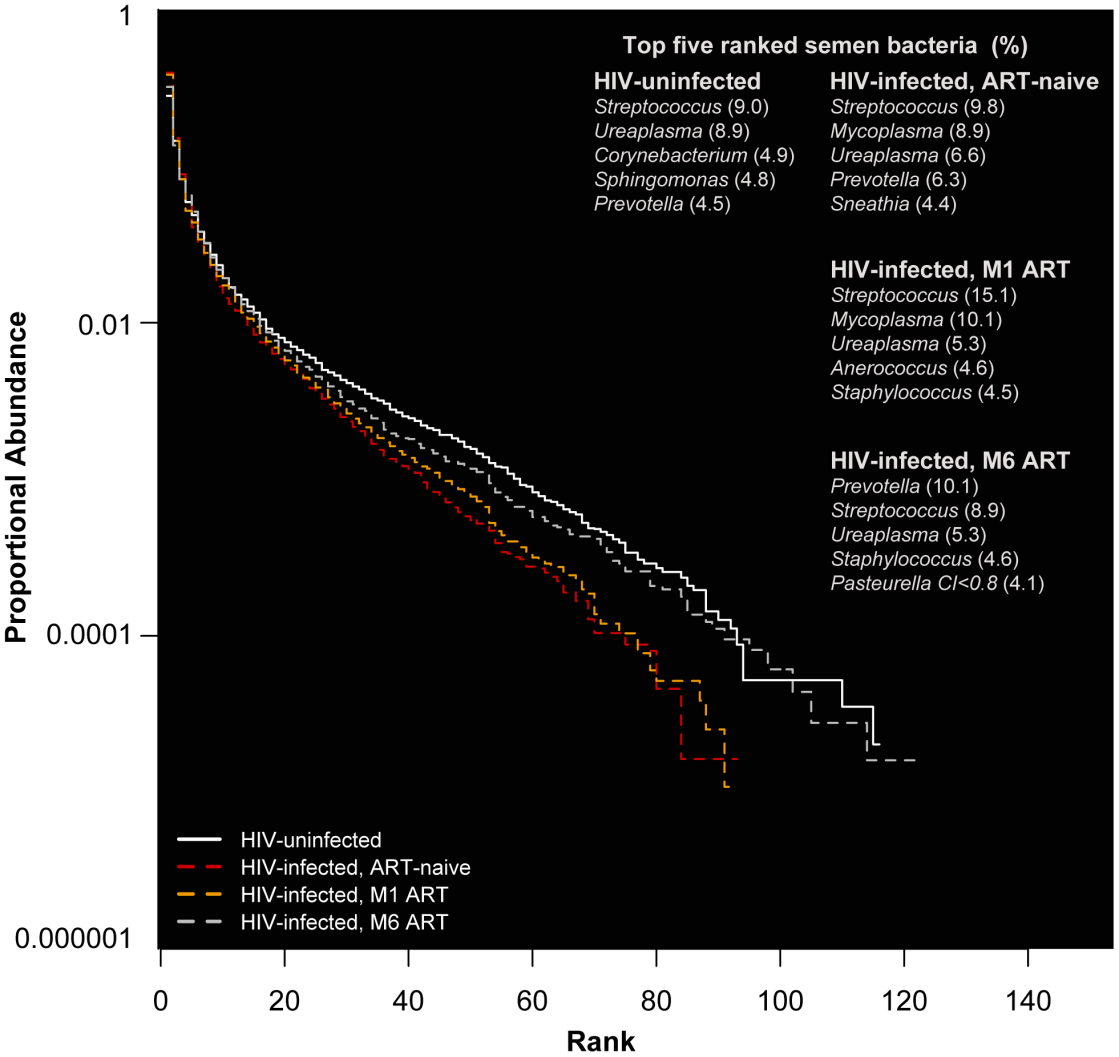
Rank abundance and the five most abundant semen bacteria in uninfected versus HIV-infected men over the course of antiretroviral treatment. In this rank abundance plot, richness is the distance the plot extends along the x-axis, and evenness is low slope. Extreme dominance is a high y-intercept. HIV uninfected is both richer and more even than the ART- naïve, but by 6 months ART, the relationship converges onto the HIV uninfected. The five top-ranked semen bacteria from each group are listed, with its respective group-level proportional abundance in parenthesis.

In HIV-uninfected MSM, *Streptococcus*, *Corynebacterium*, and *Staphylococcus* were among the most prevalent and proportionally abundant semen bacteria. Other bacteria that were proportionally abundant and comprised of 1–3% of the semen microbiota included *Prevotella*, *Porphyromonas*, *Finegoldia*, *Micrococcus*, and *Actinomyces* (**Figure 1A** **,** **Table 2** **, Table S1 in Text S1**). While *Ureaplasma* was only seen in four men, it comprised of a large portion of the semen microbiome in these individuals (**Figure 1A** **,** **Table 2** **, Table S1 in Text S1**). We detected a total of 248 unique semen bacterial genera, though not all were seen in all men. On average, 71 distinct semen bacterial genera were found in each man (*SD* = 27).

**Table 2:** Prevalence and proportional abundances of the 40 most prevalent semen bacteria in uninfected men and HIV-infected men prior to initiation of antiretroviral therapy.

	HIV-uninfected (n = 22)	HIV-infected, ART-Naïve (n = 27)	HIV-uninfected (n = 22)	HIV-infected, ART-Naïve (n = 27)
	*Prevalence (%)*		*Mean Proportional Abundance (SD)*	
***Ureaplasma***	4 (18.2%)	5 (18.5%)	0.0654 (0.2157)	0.0498 (0.1763)
***Mycoplasma***	4 (18.2%)	5 (18.5%)	0.0003 (0.0012)	0.067 (0.2182)
***Streptococcus***	22 (100%)	26 (96.3%)	0.0664 (0.0873)	0.0742 (0.1577)
***Corynebacterium***	22 (100%)	27 (100%)	0.0359 (0.0791)	0.0229 (0.0369)
***Staphylococcus***	21 (95.5%)	21 (77.8%)	0.0294 (0.0536)	0.0292 (0.0443)
***Delftia***	21 (95.5%)	20 (74.1%)	0.0118 (0.0205)	0.0061 (0.0092)
***Sphingomonas***	20 (90.9%)	20 (74.1%)	0.0356 (0.0786)	0.0105 (0.0179)
***Geodermatophilaceae CI<0.80***	20 (90.9%)	14 (51.9%)	0.019 (0.0282)	0.0043 (0.0096)
***Bacillus***	19 (86.4%)	12 (44.4%)	0.0069 (0.0117)	0.0034 (0.0068)
***Rothia***	19 (86.4%)	13 (48.2%)	0.0094 (0.0278)	0.0024 (0.0036)
***Prevotella***	18 (81.8%)	21 (77.8%)	0.0331 (0.0544)	0.0474 (0.1031)
***Micrococcus***	15 (68.2%)	16 (59.3%)	0.017 (0.0301)	0.0196 (0.0703)
***Enhydrobacter***	15 (68.2%)	6 (22.2%)	0.0045 (0.0055)	0.0008 (0.002)
***Acinetobacter***	14 (63.6%)	14 (51.9%)	0.0141 (0.0234)	0.0027 (0.0038)
***Finegoldia***	14 (63.6%)	14 (51.9%)	0.021 (0.0488)	0.0216 (0.0485)
***Actinomyces***	14 (63.6%)	16 (59.3%)	0.0166 (0.0547)	0.0086 (0.0188)
***TM7 genera incertae sedis***	14 (63.6%)	15 (55.6%)	0.0026 (0.0035)	0.0071 (0.0172)
***Neisseria***	14 (63.6%)	12 (44.4%)	0.0055 (0.0163)	0.0052 (0.0147)
***Anaerococcus***	13 (59.1%)	9 (33.3%)	0.0084 (0.0196)	0.0143 (0.0492)
***Haemophilus***	13 (59.1%)	16 (59.3%)	0.0074 (0.0182)	0.0141 (0.0232)
***Porphyromonas***	13 (59.1%)	10 (37.0%)	0.0176 (0.0458)	0.0133 (0.06)
***Gemella***	12 (54.6%)	10 (37.0%)	0.0029 (0.0046)	0.0026 (0.006)
***Flavobacterium***	12 (54.6%)	8 (29.6%)	0.0022 (0.004)	0.0016 (0.0041)
***Massilia***	12 (54.6%)	4 (14.8%)	0.0055 (0.0096)	0.0012 (0.0032)
***Peptoniphilus***	11 (50.0%)	11 (40.7%)	0.0091 (0.0294)	0.0125 (0.031)
***Sphingomonas CI<0.80***	11 (50.0%)	7 (25.9%)	0.0031 (0.007)	0.0005 (0.0008)
***Microbacterium***	11 (50.0%)	12 (44.4%)	0.002 (0.0039)	0.0012 (0.0038)
***Pseudonocardia***	11 (50.0%)	1 (3.7%)	0.0017 (0.0028)	0.0002 (0.0012)
***Veillonella***	10 (45.5%)	14 (51.9%)	0.0035 (0.0068)	0.0307 (0.0705)
***Haemophilus CI<0.80***	10 (45.5%)	17 (63.0%)	0.0021 (0.0035)	0.0094 (0.0258)
***Singulisphaera***	10 (45.5%)	10 (37.0%)	0.0042 (0.0091)	0.015 (0.0404)
***Marmoricola***	10 (45.5%)	6 (22.2%)	0.0009 (0.0016)	0.0008 (0.0019)
***Cyanobacteria.GpI***	9 (40.9%)	9 (33.3%)	0.022 (0.0693)	0.0042 (0.0094)
***Pasteurella CI<0.80***	9 (40.9%)	12 (44.4%)	0.0158 (0.0442)	0.0165 (0.0415)
***Deinococcus***	9 (40.9%)	10 (37.0%)	0.0066 (0.0253)	0.0009 (0.0019)
***Marmoricola CI<0.80***	9 (40.9%)	4 (14.8%)	0.0014 (0.0031)	0.0018 (0.0066)
***Campylobacter***	9 (40.9%)	10 (37.0%)	0.0173 (0.0444)	0.0238 (0.0705)
***Paracoccus***	8 (36.4%)	13 (48.2%)	0.0032 (0.0078)	0.0197 (0.0788)
***Corynebacteriaceae CI<0.80***	8 (36.4%)	5 (18.5%)	0.0009 (0.0018)	0.0004 (0.0011)
***Bacillus CI<0.80***	8 (36.4%)	4 (14.8%)	0.0005 (0.0009)	0.0003 (0.0005)

There was substantial variation in semen microbiome (**Figure S2**) and semen bacterial load among the HIV-uninfected men (*Mean* = 5.05 log_10_ 16S rRNA gene copies per ml of semen, *Range* = <3.6 log_10_–7.11 log_10_). Despite this, we found that there was a significant correlation between the semen microbiota composition and semen bacterial load within an individual (*r* = 0.31, *p* = 0.01) (**Figure S3A**).

### Impact of HIV and antiretroviral treatment on the semen microbiome and bacterial load

HIV infection did not significantly impact semen microbiome, but the restoration of immunity by ART modified the relationship between semen microbiome and CD4+ T-cell count. The semen bacterial load of treatment-naïve, HIV-infected men (*Mean* = 4.6 log_10_ copies/ml, *Range* = <3.6 log_10_–6.52 log_10_ copies/ml) did not differ significantly from uninfected controls *Mean* = 5.05 log_10_ 16S rRNA gene copies per ml of semen, *Range* = <3.6 log_10_–7.11 log_10_; *p = *0.13). Likewise, the initiation of ART did not significantly impact semen bacterial load.

*Streptococcus* was the most common bacterium in semen, irrespective of HIV infection status **(** **Table 2** **, Table S1 in Text S1**). The semen microbiome composition had several distinct features in association with HIV infection (**Table S2 in Text S1**), but its overall composition was not affected significantly (PerMANOVA *p* = 0.30). In uninfected controls, *Ureaplasma* was the dominant Mollicute, with *Ureaplasma parvum* was the primary sequence type. *Mycoplasma* dominated in HIV-infected men, with an average proportional abundance of 6.7%, in contrast to 0.03% in uninfected controls. With ART, the proportional abundance of *Mycoplasma* decreased over time in the HIV-infected men (**Figure 1B–D** **, Table S1 in Text S1**). Other semen bacteria also decreased after HIV infection, including *Pseudonocardia*, *Enhydrobacter*, *Bifidobacterium*, among others, while *Peptostreptococcus* and *Actinomycetospora* were uniquely prevalent and abundant in the ART-naïve men (**Table S2 in Text S1**).

A new relationship between the semen microbiome and host immunity emerged after ART. Prior to ART, the semen microbiome composition, represented by the ordination score matrix from non-metric multidimensional scaling, was correlated with semen bacterial load (*r* = 0.45, *p* = 0.004) (**Figure S3A**). However, after month one of ART, this initial correlation was lost (**Figure S3A**), and after six months of ART, the semen microbiome composition became correlated with the CD4+ T-cell count (*r* = 0.24, *p* = 0.03) (**Figure S3B**). Thus, as ART restored immunity, individuals with higher CD4+ T-cell counts had significantly different semen microbiome from those with lower CD4+ T-cell counts. This suggests that restored CD4-mediated immunity might shape the semen microbiome among HIV-infected individuals (**Figure S3**).

### HIV infection and the semen microbiome biodiversity

**Figure 2:**
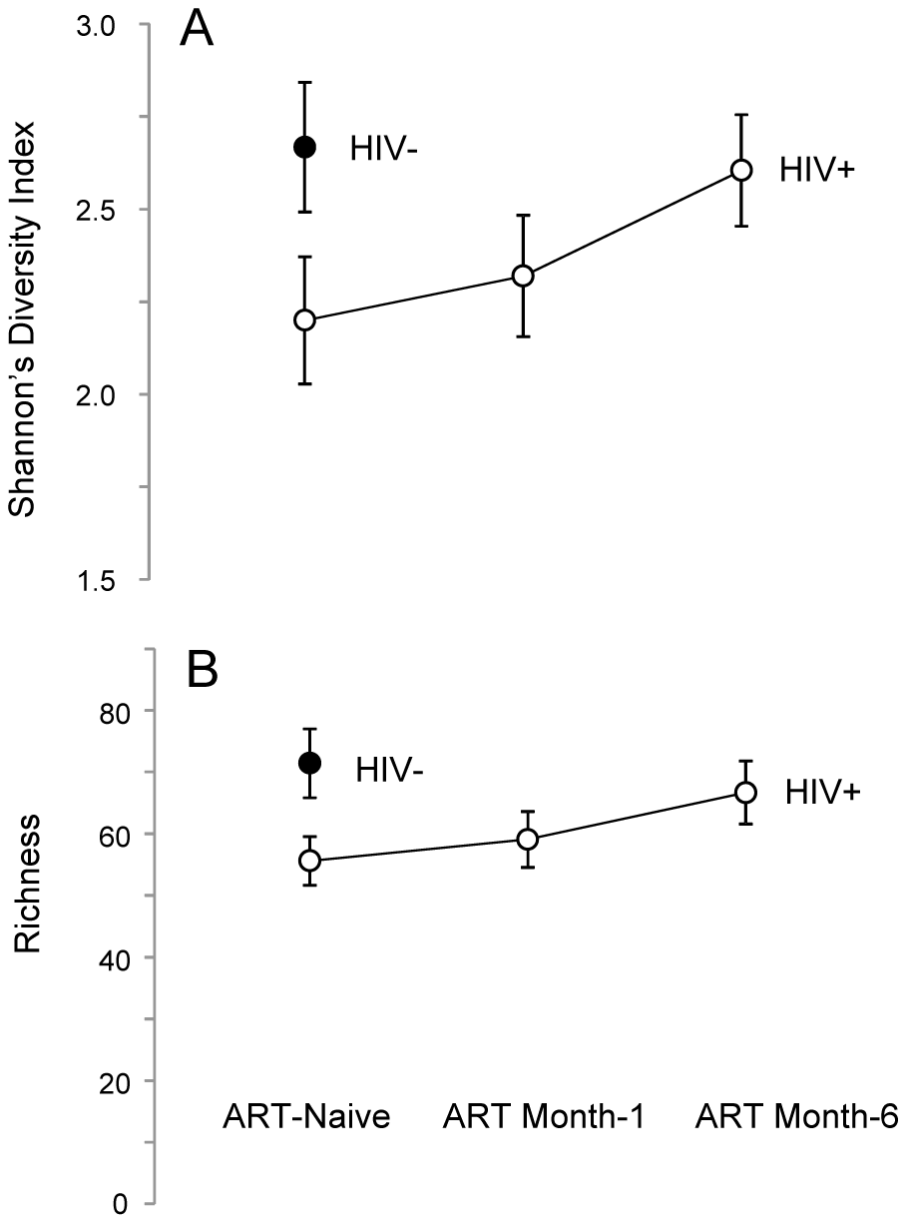
Semen microbiome biodiversity in uninfected versus HIV-infected men over the course of antiretroviral treatment. In this set of two plots (*Panels A–B*), the solid circles represent HIV-uninfected men, whereas open circles represent HIV-infected men. Panel A depicts the higher richness (*i.e.*, greater number of unique bacterial types) of the semen microbiome in HIV-uninfected men, as well as the restoration of richness over the period of six months on ART. A similar trend was seen in semen microbiome diversity, as shown in Panel B.

Another effect of ART was the reconstitution of semen microbiome biodiversity, as illustrated by microbiome diversity (*D*), richness (*S*), and evenness (*E*). Untreated HIV-infected men had a significantly lower semen microbiome diversity (*D_Mean_* = −0.5, *95% CI* = [−0.03, −0.95]) (**Figure 2A**), representing an average loss of nearly 16 genera (*S_Mean_* = −15.8, *95% CI* = [−2.8, −28.4]) (**Figure 2B**). However, one aspect of semen microbiome biodiversity that remained unchanged after HIV infection was the equitability of bacterial constituents (E*_Mean_* = −0.0006, *95% CI* = [−0.007, 0.005]).

As ART restored CD4+ T-cell counts, the number of unique semen bacteria increased, (Repeated Measures ANOVA, *p* = 0.027), accompanied by restoration of semen microbiome diversity (Repeated Measures ANOVA, *p* = 0.081) (**Figure 2A**). After 6 months of ART, the semen richness increased by 11 genera (95% CI, 3.0 to 19.7) and the Shannon Diversity index had increased by 0.41 (95% CI 0.04 to 0.75); at this point, the semen microbiome biodiversity became statistically indistinguishable from that of uninfected controls (**Figure 2A–B**). This restitution of microbiome biodiversity with improved host immunity further supports the role of host immunity in regulating bacteria in the semen.

Notably, the increase in biodiversity was not driven by the reappearance by bacteria that defined the HIV-uninfected state. Rather, distinct bacteria, such as *Pedobacter*, showed a significant proportional increase at 6 months of ART (**Table S3 in Text S1**). In contrast, semen bacteria associated with HIV-uninfected men showed only modest increase, with *Enhydrobacter* peaking after one month of ART and *Pseudonocardia* peaking six months of ART. *Bifidobacterium* showed no appreciable increase (**Figure S4, Table S2 in Text S1**).

### Semen bacterial load and cytokine levels prior to ART

We hypothesized that, in HIV-infected men, the semen bacterial load would correlate with pro-inflammatory cytokine/chemokine levels in the semen. Our data supported the hypothesis, revealing a markedly reorganized relationship between semen bacterial load and cytokines after HIV infection. In uninfected controls, our data showed no correlation between semen bacterial load and cytokine levels. However, in HIV-infected men, seven out of the 13 semen cytokines correlated with semen bacterial load, and specifically, for each 10% increase in semen bacterial load, there was a corresponding 2% increase in MIG (*p* = 0.03), IL-10 (*p* = 0.02), IL-6 (*p* = 0.024), IP-10 (*p*  = 0.03), IL-17 (*p* = 0.03) and TNF-α (*p* = 0.009) and a 3% increase in IL-1b (*p* = 0.002).

Using principal component analysis, we found that the seven semen cytokines could be collapsed into three orthogonal (independent) components, with the first component being driven by IL-6, IL-17, IP10, and TNF-α, the second component by IL-1b, and third component by MIG. IL-10 contributed to all three (**Table S4 in Text S1**). Among the three components, only the second (IL-1b) correlated significantly with semen HIV RNA VL (*r^2^* = 0.18, *p* = 0.02).

The correlations between IL-1b and both semen bacterial load and semen HIV RNA VL could not be explained by natural patterns in cytokine variation. There was considerable heterogeneity in semen cytokine levels, with an inter-individual range for most cytokines exceeding 2 log_10_ pg/ml, and median levels ranging from 0.3 log_10_ pg/ml (IL-10) to 3.8 log_10_ pg/ml (IP-10). However, neither the degree of variability nor the median levels of cytokines related to semen bacterial or viral load.

Further analysis did not reveal significant associations between specific semen bacteria and local semen immunology, beyond the associations already described with semen bacterial load. While the total semen bacterial load correlated with pro-inflammatory cytokine levels in the semen of HIV-infected men, this finding reflects the individualized nature of the semen microbiome in both HIV-infected and uninfected men.

### Semen bacterial load and HIV viral load prior to in ART-naïve men

**Figure 3:**
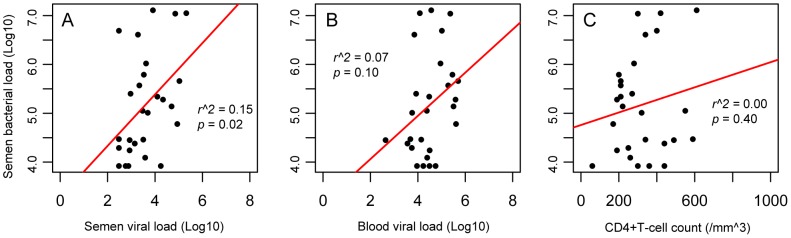
Correlation of semen bacterial load with CD4+ T-cell counts and viral loads in the semen and blood of HIV-infected men prior to antiretroviral treatment. Our data showed multiple correlations between HIV loads and CD4+ counts, including the correlation of semen and blood viral loads and of blood CD4+ T-cell counts and viral load (Figure S1). However, we found that the significant correlation between semen bacterial load with semen viral load was unique (Panel A), and the semen bacterial load showed no such correlation with either the blood viral load (Panel B) or CD4+ T-cell counts (Panel C), suggesting that the correlation between bacterial and viral loads in the semen might be caused by localized mechanisms.

We also found that semen bacterial load was directly and significantly correlated with semen HIV RNA levels prior to ART initiation. Specifically, with each 10% increase in semen bacterial load, semen viral load increased by 3% (*r^2^* = 0.15, *p* = 0.02) (**Figure 3A**). In contrast, the semen bacterial load was not correlated with blood viral load (*r^2^* = 0.07, *p* = 0.10) (**Figure 3B**) or blood CD4+ T-cell count (*r^2^* = 0.01, *p* = 0.28) (**Figure 3C**). The rapid drop in semen viral load after ART initiation did not permit assessment of the association between semen bacterial load and HIV shedding in men treated with ART.

Since the semen HIV RNA VL was also positively correlated with the blood VL, a multivariable logistic regression model was used to assess the independent association of semen HIV levels with blood VL and total semen bacterial load. To increase our statistical power in the multivariate model, we assessed the total bacterial load in an additional 22 HIV-infected, ART-naïve MSM participants (median CD4+ T count 410 cells/mm^3^, blood HIV-1 RNA load 43,008 copies/ml, and semen HIV-1 RNA load 6,168 copies/ml). In this larger dataset, the semen RNA VL remained strongly associated with both semen bacterial load and blood HIV viral load on univariate analysis (*r^2^* = 0.45, *p* = 0.001 and *r^2^* = 0.37, *p* = 0.006, respectively). The total semen bacterial load remained independently associated with semen VL in multivariate logistic regression (*t* = 2.80, *p* = 0.007), while the association with blood VL became borderline significant (*t* = 2.00, *p* = 0.05).

## Discussion

The semen HIV RNA VL is a crucial measure of the infectiousness of genital secretions in HIV-infected men. We had hypothesized that microbiome-driven changes in semen immunology could impact the semen VL. In this study, we found significant links between the semen bacterial load, the level of semen pro-inflammatory cytokines and the semen HIV RNA VL. In addition, our data demonstrated that HIV RNA shedding in the semen correlated with increases in semen bacterial load and IL-1b.

Since men who have sex with other men (MSM) are at particularly high risk in the North American HIV epidemic, our work focused on this population. Semen is a composite of spermatozoa and secretions from the prostate, seminal vesicles, and epididymis, and the precise sources of semen bacteria have not yet been pinpointed. In particular, it is not clear whether these bacteria are acquired from a partner during sexual intercourse or originate from other host sites such as the urinary tract, gut or foreskin. Earlier male urogenital bacterial studies have focused on heterosexual men [18], [19], [22], where vaginal bacteria such as *Lactobacillus*, *Prevotella*, *Porphyromonas*, *Veillonella* have been shown in their semen, coronal sulcus, urethral, and urine [19], [23]–[25]. We observed that bacteria in the semen of MSM overlapped with those previously described in the vagina, including *Prevotella* and *Mycoplasma*; the latter, as well as *Ureaplasma*, has been implicated in male infertility [26].

Our findings of Mollicutes in the semen of MSM, together with previous reports in heterosexual men, suggest that semen is a source for Mollicutes and may shed light on the directionality of their sharing in heterosexual couples. Mollicutes and another semen bacteria, *Streptococcus*, are primarily associated with moist, mucosal surfaces and are rare in the penile coronal sulcus [25]. In contrast, *Corynebacterium* and *Staphylococcus* were common in the semen of MSM and are also known inhabitants of skin and in the coronal sulcus of circumcised men [25]. Thus, our findings show that semen likely contains bacteria that originate from multiple sites within the male genital tract.

There are a few limitations to our study. We did not collect sexual behavior data, which would have allowed for interesting additional analyses. However, it is unlikely that the alterations in the semen microbiome diversity of HIV-infected men were due to changes in sexual behavior, for two reasons. First, with the exception of *Prevotella*, which can be found in multiple body sites (including penile, vaginal, oral cavity, and gut) but can only have limited effect on biodiversity as a single genus, we found a limited overlap among semen, oropharyngeal, and gastrointestinal bacteria. Second, the increase in bacterial diversity was observed shortly after ART initiation, during a period when patients had enhanced medical follow-up and extensive counseling against unprotected sex due to our own observations of isolated HIV shedding [27]. The methods used for semen processing were optimized for detection of HIV in semen, and should be further refined for future semen microbiome research. Specifically, our use of a high centrifugation speed (850 g) might have differentially pelleted bacterial species in semen, and our attempts to quantify bacteria in the semen pellet were unsuccessful due to high levels of human DNA that interfered with our method of bacterial DNA detection. Therefore, future semen microbiome studies should consider lower centrifugation speeds and methods to differentially extract bacterial DNA from semen. The volume of semen should also be recorded to enhance semen bacterial load analysis. Lastly, since the hand and penile skin contain bacteria that could contaminate semen samples, participants should be instructed to clean their hands and penis prior to providing a semen sample.

We found the biodiversity of semen microbiome to be low as compared to that previously described at other body sites [28] and further reduced by HIV infection. From an ecological perspective, this finding could reflect increased inter-bacterial competition as a result of impaired local host immunity during HIV infection. After ART initiation, the semen microbiome biodiversity was restored: together with the correlation between CD4+ T-cell counts and semen microbiome composition after ART, this suggests that local host immunity plays a role in shaping the semen microbiome. However, it is difficult to predict the relevance of this reduced semen microbiome diversity to health outcomes in the host. While reduced microbiome diversity has been associated with inflammation and negative health outcomes in the gastrointestinal tract [29], [30], reductions in the penis microbiome diversity after male circumcision have been hypothesized to play a beneficial role in the procedure's protective effects against HIV acquisition [25].

Previous studies have linked elevated semen VL to sexual practices such as unprotected insertive anal sex [7], which could introduce gastrointestinal bacteria into the male urogenital compartment and semen. Likewise, the finding of persistent semen HIV shedding early after the initiation of effective ART suggest that compartmentalized, non-treatment related factors such as the semen microbiome could play a role in local shedding of HIV [27]. In our study, all participants improved with treatment, as evidenced by the rapid decreases in blood and semen VL. While this highlights the effectiveness of ART, it will be important to investigate the potential role of semen bacteria in mucosal inflammation and the semen VL among ART-naïve men, particularly those whose semen VL is disproportionately higher than that in blood [31], [32]. It will also be important to evaluate a possible role of semen bacteria in those men who maintain a high semen VL despite effective ART [27]. Understanding the role of semen bacteria in these contexts could shed new light on HIV sexual transmission and lead to novel avenues for prevention.

Our study showed that the semen bacterial load in HIV-infected ART-naïve men was correlated with the semen HIV VL and with the levels of several pro-inflammatory cytokines in the semen; among these, semen IL-1b levels were also correlated with semen HIV VL. This suggests that semen bacteria, in the context of untreated HIV infection, may induce a local inflammatory milieu and drive increased HIV shedding and transmission, although the restoration of reduced semen bacterial diversity post-ART implies a reciprocal role for host immunity in shaping the semen microbiome. While delineating the directionality and causality of these complex relationships will require further studies, our data support the hypothesis that semen bacteria play a role in local inflammation and HIV shedding, and is a possible target for reducing HIV transmission.

## Methods

### Ethics statement

Adult MSM, age 18–65, without physical or laboratory evidence of *C. trachomatis* or *N. gonorrhoeae* or history of *T.pallidum* infection were eligible to participate in the study. HIV-infected, treatment-naïve individuals were enrolled through the Maple Leaf Medical Clinic in Toronto, Canada. HIV-uninfected participants were volunteers recruited from the staff and student body of University of Toronto. All study participants provided written informed consent, and the study was approved by the Research Ethics Board at the University of Toronto (Toronto, Canada) (protocol #26946) and by TGen's IRB of record, the Western Institutional Review Board (protocol # 20081375).

### Study design

This observational study of men who have sex with men (MSM) in Toronto, Ontario, Canada examined the changes in the semen microbiome associated with HIV infection and treatment with antiretroviral therapy (ART) using a single semen specimen from HIV-uninfected MSM as controls and paired blood and semen samples form HIV-infected MSM prior to treatment and at months 1 and 6 after initiation of standard-of-care antiretroviral therapy.

### Sample collection and initial processing

Participants were instructed to abstain from intercourse and masturbation for 48 hours prior to sample collection. Semen samples were collected by masturbation into 10 mL sterile RPMI 1640 containing 100 U/mL penicillin and 100 mg/mL streptomycin (Gibco). Seminal plasma was isolated by centrifugation at 850 g for 10 minutes. Blood samples were collected directly into acid citrate dextran and the blood plasma was isolated by ficoll density gradient centrifugation at 500 g for 25 minutes. All samples were stored at −80°C until analysis.

### *Chlamydia trachomatis*, *Neisseria gonorrhoeae*, and *Treponema pallidum* screening

Laboratory diagnosis of *C. trachomatis* or *N. gonorrhoeae* urethritis was performed using seminal plasma by urine nucleic acid amplification (Amplicor CT/NG assay, Roche Diagnostics, QC, Canada). *T. pallidum* infection was determined by serology (RPR; rapid plasma regain, BioRad, QC, Canada).

### Blood and semen viral load quantification

Blood and semen HIV-1 RNA concentrations were measured using the Versant HIV RNA 3.0 assay (bDNA Bayer Diagnostics, Puteaux Cedex, France) in the Mount Sinai Hospital Department of Microbiology. Correction for semen dilution was calculated based on an average ejaculate volume of 2 mL, as described previously [33].

### Semen bacterial cell lysis and nucleic acid purification

We lysed 500 µl of thawed seminal plasma using a combination of chemical and mechanical methods, purified using Qiagen AllPrep DNA/RNA Mini Kit (Qiagen, Valencia, CA, USA), and the DNA was eluted in 100 µl of buffer EB, while RNA was eluted in 50 µl. Reverse transcription was performed using qScript cDNA SuperMix following the manufacturer's instructions (Quanta Biosciences, Geithersburg, MD, USA). Additional details can be found in the Supplementary File.

### Semen bacterial load quantification and 16S rRNA-based pyrosequencing analysis

Using the DNA fraction, we quantified the bacterial load, measured as the bacterial 16S rRNA gene copy per ml of seminal plasma using a broad-coverage qPCR assay [34]. Using the cDNA fraction, we generated barcoded V3–V6 amplicons for pyrosequencing and processed the resultant sequence data as previously described [25]. Additional details can be found in the Supplementary File.

Pyrosequencing yielded a total of 162,998 16S rRNA gene sequences at ≥80% bootstrap confidence level after taxonomic groups with fewer than ten sequences in the full dataset. For sequence types that could not be further classified at ≥80% bootstrap confidence level past a higher taxonomic level (e.g., Clostridiales), they were specified as “Unclassified” (e.g., Unclassified Clostridiales).

### Seminal plasma cytokine quantification

We analyzed the cytokine/chemokine levels in 21 HIV-uninfected participants and in 25 HIV-infected participants at baseline. Cytokine and chemokine levels in seminal plasma were measured using the Meso Scale Discovery SECTOR Imager 2400 (Meso Scale Discovery, Rockville, MD) multiplexing system following manufacturer's instructions. Using samples collected at 1∶6 dilution, we measured 13 cytokines and chemokines including interleukin (IL)-1α, IL-8, monocyte chemotactic protein-1 (MCP-1), Monokine induced by gamma interferon (MIG), Macrophage inflammatory protein-3 (MIP-3α), Regulated And Normal T-cell Expressed and Secreted (RANTES), IL-10, IL-17, IL-1β, IL-6, Interferon gamma-induced protein 10 (IP-10), MIP-1β and tumor necrosis Factor alpha (TNF-α). Log_10_-transformed cytokine levels were used in subsequent analysis.

### HIV infection parameters in the infected group

We examined the change in CD4+ T-cell counts and semen and blood viral loads in HIV-infected men at three time points. Correlation between these three infection parameters were further examined by linear regression in R version 2.13.1 [35]. All subsequent analyses were performed in R, unless otherwise specified.

### Microbiome analysis

We analyzed semen microbiome using genus-level data and two primary metrics: prevalence and proportional abundance. Specifically, prevalence was calculated as: *(Total number of participants with more than two sequences for the genus A in group X)/(Total number of participants in group X).* Proportional abundance as: *(Number of sequences assigned to the genus A in participant A)/(Total number of sequences from participant A).*

After eliminating rare taxa detected at less than 1.5% proportional abundance on a per-sample basis, we determined the prevalence and proportional abundance of semen bacteria in the uninfected controls and in HIV-infected MSM at three time points. We compared top-ranked semen bacteria in each group based on the sum of per-individual proportional abundance [36].

We visualized microbiome data using heatmap and non-metric multidimensional scaling based on Bray-Curtis distance. Specifically, we converted the microbiome data matrix (measured in semen bacteria proportional abundances) to a matrix based on Bray-Curtis distance. We extracted the most informative components of the distance matrix by ordination (non-metric multidimensional scaling), and then fitted the variables of interest onto the resultant ordination score matrix. Using this approach, we assessed correlations between the semen microbiome composition and semen bacterial load (log_10_), semen and blood viral loads, and CD4+/CD8+ counts, we further examined these correlations by vector fitting each variable as a vector or factor using the ordinated ordination score matrix. We also evaluated if HIV infection status has a global effect on semen microbiome composition by permutational ANOVA [36]. A significance level of α = 0.05 was used.

We evaluated the effects of HIV infection and ART on the biodiversity of semen microbiome based on Diversity (*D*), calculated as *D* = Shannon diversity index, evenness (*E*), calculated as *E* = *D*/log(*S*), and *S* = richness [37]. Whereas richness represents the total number of unique taxa that have been detected, evenness reflects the dominance by many (*i.e.*, high evenness) versus few (*i.e.*, low evenness) taxa. The effect size of HIV infection and of ART on each biodiversity metric, including the associated 95% CIs, was estimated by bootstrapping. Statistical significance of the change after ART was determined by repeated measures ANOVA.

We utilized indicator species analysis to identify the semen bacteria that differed between uninfected men and HIV-infected, untreated men. A significance level of α = 0.10 was used [38]. We also extracted all Mollicute sequences from all positive samples and examined the sequences using the SeqMan software (DNASTAR Inc., Madison, WI, USA).

### Bacterial load analysis

The log_10_-transformed bacterial load data was used for all statistical analyses. We evaluated the association of semen bacterial load with HIV infection status and ART using the Wilcoxon ranked-sum and signed-rank test, respectively. We further assessed the relationship between semen bacterial load, the blood and semen viral loads and CD4+ T-cell counts by univariate linear regression and multivariate logistic models. Significance of α = 0.05 was used.

### Cytokine analysis

We built univariate linear regression models to examine the correlation between semen cytokines and semen bacterial load using α = 0.05. To address potential covariation among cytokines that correlated significantly with semen bacterial load, we applied principal component analysis with varimax rotation using the *psych* package Version 1.3.10 in R [39]. The resultant three-component solution explained 93% of total variance. Lastly, we examined the correlation of each component with semen viral load by linear regression again using α = 0.05.

## Supporting Information

Figure S1 **Correlation of CD4+ T-cell counts with semen and blood viral loads.** Prior to antiretroviral therapy (ART), the CD4+ T-cell counts were moderately correlated with blood viral load (*r^2^* = 0.27, *p* = 0.003), but not with the viral load in semen. There was also a moderate correlation between the blood and the semen viral load (*r^2^* = 0.27, *p* = 0.003) (*Panel A*), but these correlation did not persist after one (*Panels B*) or six months of ART (*Panel C*).
(TIF)

Figure S2 **Heatmap visualization of semen microbiome in HIV-uninfected (n = 22) and HIV-infected men (n = 27) based on proportional abundance.** Each column in this heatmap visualization shows the semen microbiome in each sample, including in HIV-infected men over the course of their treatment. Along each row, the proportional abundance each semen bacterial genus (*e.g.*, *Propionibacterium*) is shown and can be interpreted using the annotated color-coding key (*right*, color bar), which denotes the color for its respective proportional abundance. The heatmap shows a wide range of semen bacteria, but few are more than 25% proportional abundant in a given sample.
(TIF)

Figure S3 **Semen microbiome composition and its correlation with semen bacterial load and CD4+ T-cell counts in HIV-uninfected (n = 22) and HIV-infected men (n = 27).** In this set of non-metric multidimensional scaling plots (*Panels A–B*), each data point represents the full semen microbiome of a single sample. Panels A and B have the same background nMDS plot, while in Panel A, the semen bacterial load (log10-transformed) from each group was fitted as vector and in Panel B, the CD4+ count was fitted. The corresponding R-square and p-value for each vector in each group is as shown. The semen microbiome in HIV-uninfected men was correlated with semen bacterial load, and this correlation was also seen in HIV-infected men prior to antiretroviral treatment (*Panel A*). However, after six months of treatment, CD4+ T-cell counts became significantly correlated with the semen microbiome (*Panel B*). There was no overall composition difference between the semen microbiome of HIV-uninfected men from men who are infected by HIV, as shown by the overlapping 95% confidence interval ellipses (*Panel A–B*).
(TIF)

Figure S4 **Changes in specific semen bacteria after one and six months of ART.** The semen bacterial changes after ART did not involve those that were unique to HIV-uninfected men. *Pedobacter* increased significantly in HIV-infected men after ART (*Panel A*). Whereas among semen bacteria that were unique to HIV-uninfected men, *Enhydrobacter* showed peaking after one month of ART (*Panel B*) and *Pseudonocardia* peaking six months of ART (*Panel C*). *Bifidobacterium* showed no appreciable increase (*Panel D*).
(TIF)

Text S1 **Supporting information.** This file includes four supporting information tables. Table S1. Prevalence and proportional abundances of semen bacteria in HIV-uninfected men and in HIV-infected men prior to and after antiretroviral therapy. Table S2. Results from the indicator analysis showing semen bacterial genera based on HIV infection status. Table S3. Semen bacteria unique in HIV-infected men after six months of antiretroviral treatment. Table S4. Loadings from the principal component analysis of pro-inflammatory cytokines that correlated with semen bacterial load.
(DOCX)
