# Supplementary figures and images for: The Semen Microbiome and Its Relationship with Local Immunology and Viral Load in HIV Infection
Source: PLoS Pathog. 2014 Jul 24;10(7):e1004262. doi: 10.1371/journal.ppat.1004262 (PMC4110035; doi:10.1371/journal.ppat.1004262)

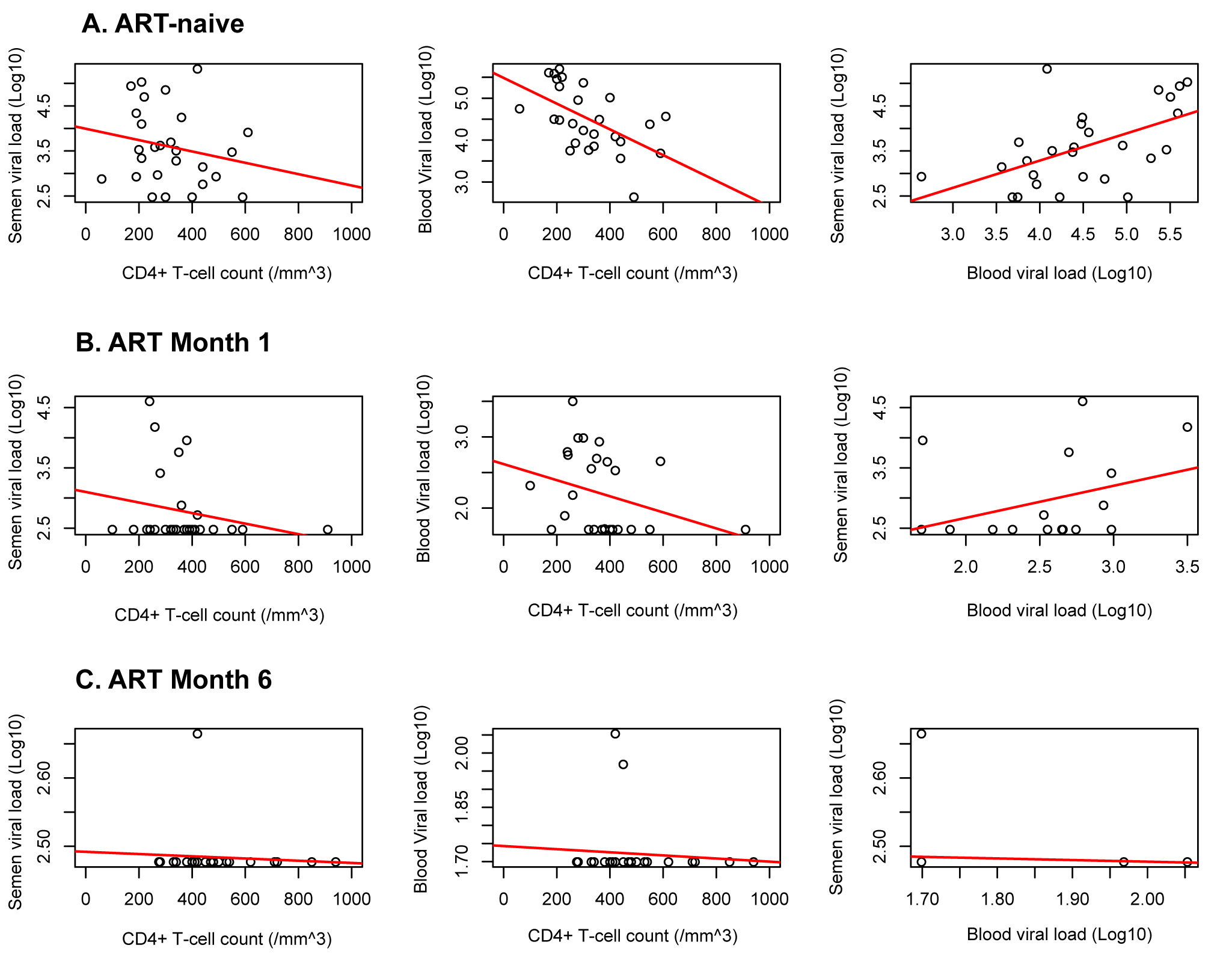

Supplement: Figure S1 — Correlation of CD4+ T-cell counts with semen and blood viral loads. Prior to antiretroviral therapy (ART), the CD4+ T-cell counts were moderately correlated with blood viral load (r2 = 0.27, p = 0.003), but not with the viral load in semen. There was also a moderate correlation between the blood and the semen viral load (r2 = 0.27, p = 0.003) (Panel A), but these correlation did not persist after one (Panels B) or six months of ART (Panel C). (TIF) [file ppat.1004262.s001.tif]

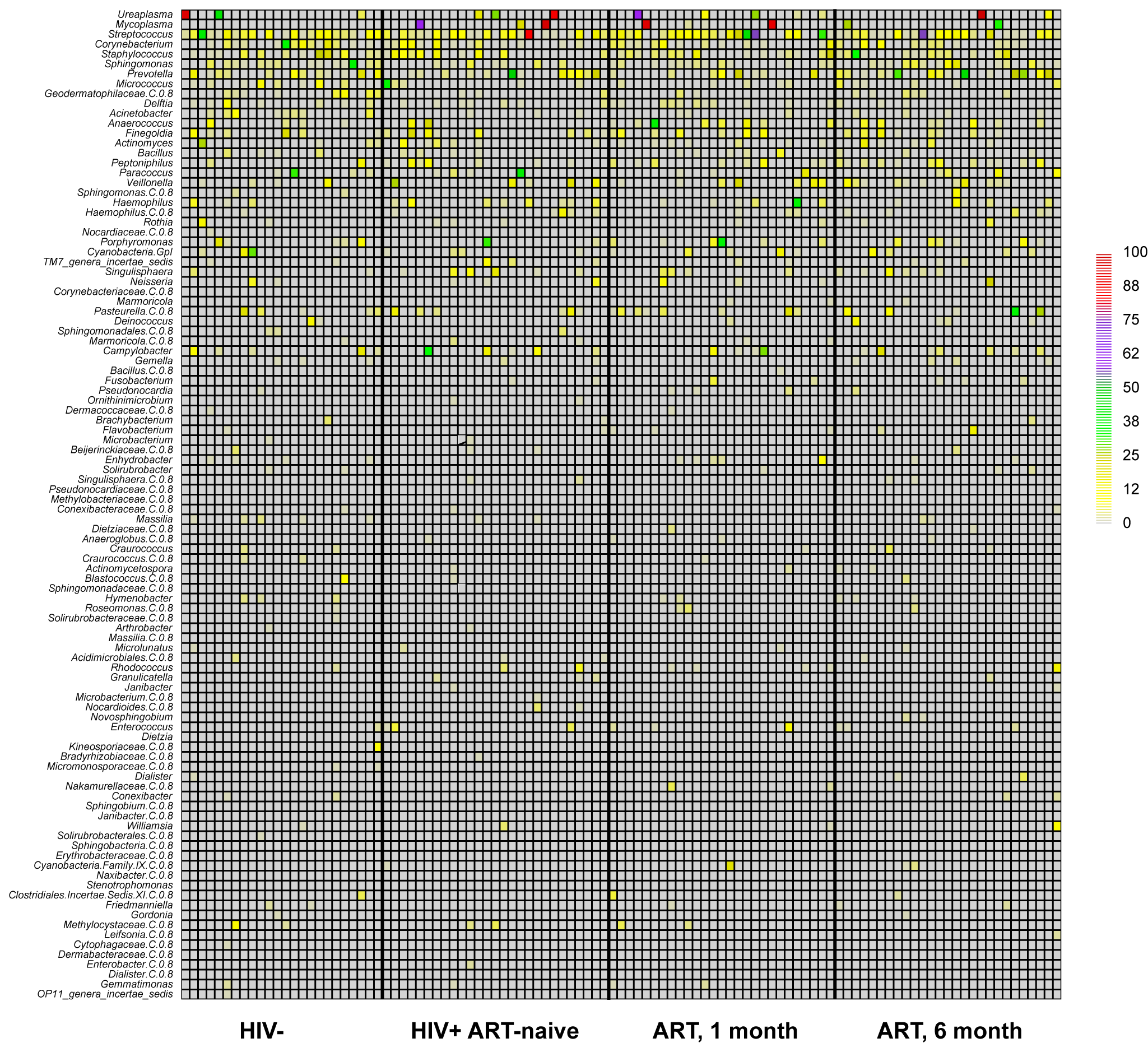

Supplement: Figure S2 — Heatmap visualization of semen microbiome in HIV-uninfected (n = 22) and HIV-infected men (n = 27) based on proportional abundance. Each column in this heatmap visualization shows the semen microbiome in each sample, including in HIV-infected men over the course of their treatment. Along each row, the proportional abundance each semen bacterial genus (e.g., Propionibacterium) is shown and can be interpreted using the annotated color-coding key (right, color bar), which denotes the color for its respective proportional abundance. The heatmap shows a wide range of semen bacteria, but few are more than 25% proportional abundant in a given sample. (TIF) [file ppat.1004262.s002.tif]

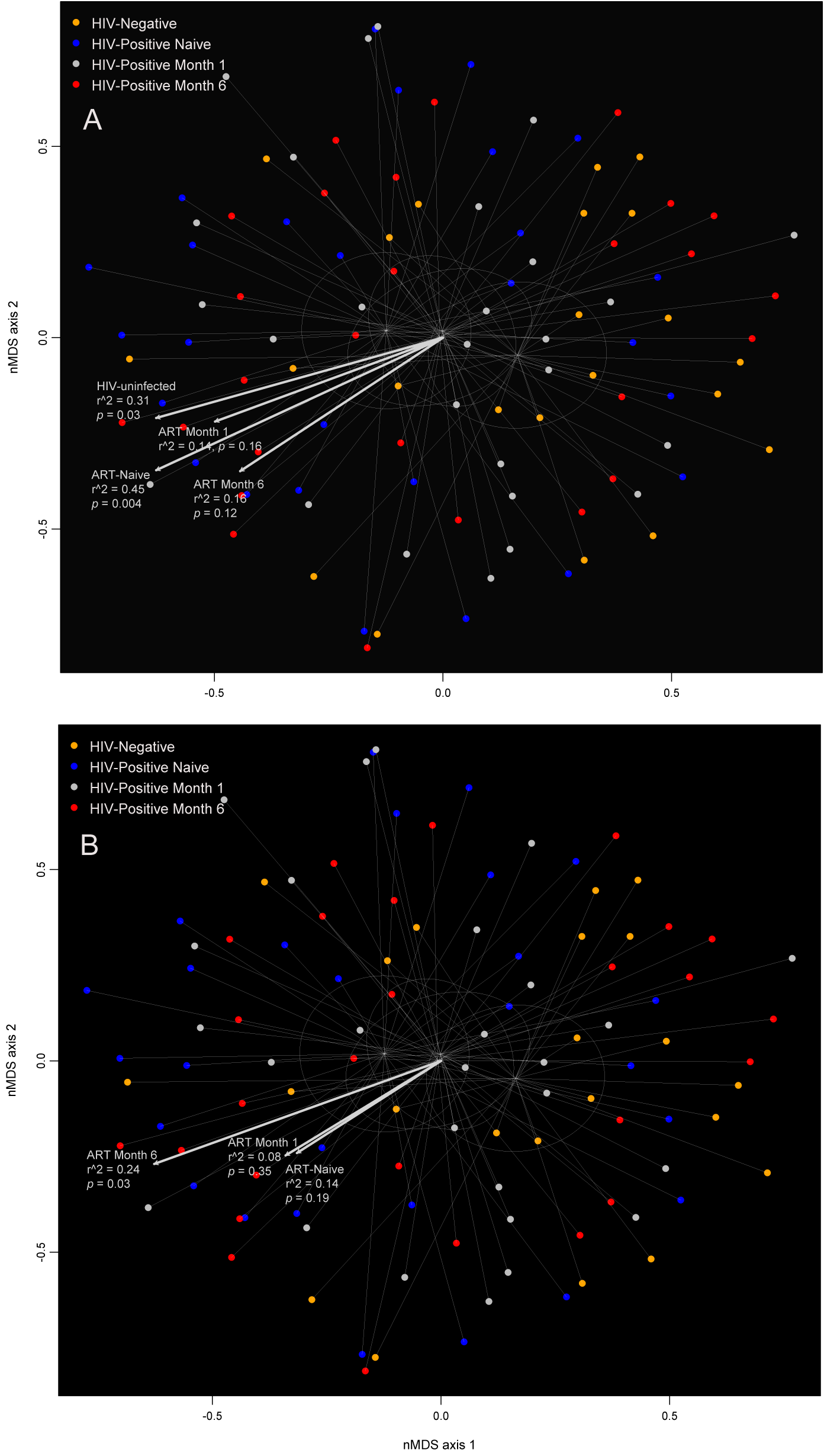

Supplement: Figure S3 — Semen microbiome composition and its correlation with semen bacterial load and CD4+ T-cell counts in HIV-uninfected (n = 22) and HIV-infected men (n = 27). In this set of non-metric multidimensional scaling plots (Panels A–B), each data point represents the full semen microbiome of a single sample. Panels A and B have the same background nMDS plot, while in Panel A, the semen bacterial load (log10-transformed) from each group was fitted as vector and in Panel B, the CD4+ count was fitted. The corresponding R-square and p-value for each vector in each group is as shown. The semen microbiome in HIV-uninfected men was correlated with semen bacterial load, and this correlation was also seen in HIV-infected men prior to antiretroviral treatment (Panel A). However, after six months of treatment, CD4+ T-cell counts became significantly correlated with the semen microbiome (Panel B). There was no overall composition difference between the semen microbiome of HIV-uninfected men from men who are infected by HIV, as shown by the overlapping 95% confidence interval ellipses (Panel A–B). (TIF) [file ppat.1004262.s003.tif]

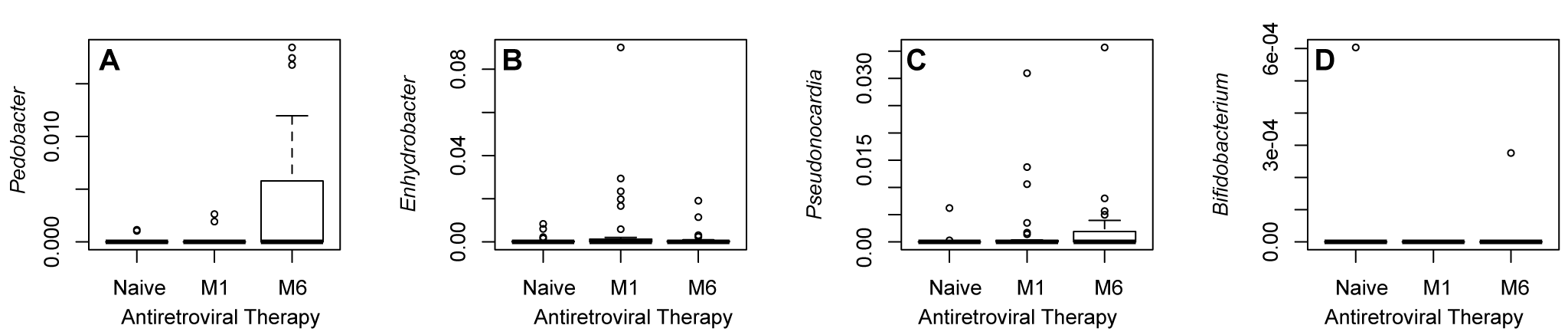

Supplement: Figure S4 — Changes in specific semen bacteria after one and six months of ART. The semen bacterial changes after ART did not involve those that were unique to HIV-uninfected men. Pedobacter increased significantly in HIV-infected men after ART (Panel A). Whereas among semen bacteria that were unique to HIV-uninfected men, Enhydrobacter showed peaking after one month of ART (Panel B) and Pseudonocardia peaking six months of ART (Panel C). Bifidobacterium showed no appreciable increase (Panel D). (TIF) [file ppat.1004262.s004.tif]
