# Supplementary material for: The Semen Microbiome and Its Relationship with Local Immunology and Viral Load in HIV Infection
Source: PLoS Pathog. 2014 Jul 24;10(7):e1004262. doi: 10.1371/journal.ppat.1004262 (PMC4110035; doi:10.1371/journal.ppat.1004262)
Supplement: Text S1 — Supporting information. This file includes four supporting information tables. Table S1. Prevalence and proportional abundances of semen bacteria in HIV-uninfected men and in HIV-infected men prior to and after antiretroviral therapy. Table S2. Results from the indicator analysis showing semen bacterial genera based on HIV infection status. Table S3. Semen bacteria unique in HIV-infected men after six months of antiretroviral treatment. Table S4. Loadings from the principal component analysis of pro-inflammatory cytokines that correlated with semen bacterial load. (DOCX) [file ppat.1004262.s005.docx]

**SUPPLEMENTARY TABLES**

**Table S1.** **Prevalence and proportional abundances of semen bacteria in HIV-uninfected men and in HIV-infected men prior to and after antiretroviral therapy.**

|  | **HIV-uninfected**  **(n =22)** | **ART Naïve**  **(n =27)** | **ART Month 1**  **(n =27)** | **ART Month 6**  **(n =27)** | **HIV-uninfected**  **(n =22)** | **ART Naïve**  **(n =27)** | **ART Month 1**  **(n =27)** | **ART Month 6**  **(n =27)** | |
| --- | --- | --- | --- | --- | --- | --- | --- | --- | --- |
|  | *Prevalence (%)* | | | | *Mean Proportional Abundance (SD)* | | | |  |
| ***Ureaplasma*** | 4 (18.2%) | 5 (18.5%) | 5 (18.5%) | 3 (11.1%) | 0.0654 (0.2157) | 0.0498 (0.1763) | 0.037 (0.1223) | 0.0399 (0.191) | |
| ***Mycoplasma*** | 4 (18.2%) | 5 (18.5%) | 4 (14.8%) | 2 (7.4%) | 0.0003 (0.0012) | 0.067 (0.2182) | 0.0702 (0.2468) | 0.0215 (0.078) | |
| ***Streptococcus*** | 22 (100%) | 26 (96.3%) | 24 (88.9%) | 26 (96.3%) | 0.0664 (0.0873) | 0.0742 (0.1577) | 0.1052 (0.1377) | 0.0668 (0.1104) | |
| ***Corynebacterium*** | 22 (100%) | 27 (100%) | 23 (85.2%) | 26 (96.3%) | 0.0359 (0.0791) | 0.0229 (0.0369) | 0.0228 (0.0354) | 0.0184 (0.0225) | |
| ***Staphylococcus*** | 21 (95.5%) | 21 (77.8%) | 24 (88.9%) | 25 (92.6%) | 0.0294 (0.0536) | 0.0292 (0.0443) | 0.0312 (0.0522) | 0.0335 (0.0721) | |
| ***Delftia*** | 21 (95.5%) | 20 (74.1%) | 21 (77.8%) | 17 (63.0%) | 0.0118 (0.0205) | 0.0061 (0.0092) | 0.0104 (0.0117) | 0.005 (0.0067) | |
| ***Sphingomonas*** | 20 (90.9%) | 20 (74.1%) | 22 (81.5%) | 20 (74.1%) | 0.0356 (0.0786) | 0.0105 (0.0179) | 0.0111 (0.0126) | 0.0249 (0.0412) | |
| ***Geodermatophilaceae CI<0.80*** | 20 (90.9%) | 14 (51.9%) | 17 (63.0%) | 17 (63.0%) | 0.019 (0.0282) | 0.0043 (0.0096) | 0.004 (0.0092) | 0.0079 (0.0122) | |
| ***Bacillus*** | 19 (86.4%) | 12 (44.4%) | 17 (63.0%) | 16 (59.3%) | 0.0069 (0.0117) | 0.0034 (0.0068) | 0.007 (0.0146) | 0.0043 (0.006) | |
| ***Rothia*** | 19 (86.4%) | 13 (48.2%) | 9 (33.3%) | 10 (37.0%) | 0.0094 (0.0278) | 0.0024 (0.0036) | 0.0023 (0.0055) | 0.0037 (0.0116) | |
| ***Prevotella*** | 18 (81.8%) | 21 (77.8%) | 16 (59.3%) | 24 (88.9%) | 0.0331 (0.0544) | 0.0474 (0.1031) | 0.0258 (0.0578) | 0.0758 (0.1208) | |
| ***Micrococcus*** | 15 (68.2%) | 16 (59.3%) | 18 (66.7%) | 18 (66.7%) | 0.017 (0.0301) | 0.0196 (0.0703) | 0.0064 (0.0086) | 0.0085 (0.0161) | |
| ***Enhydrobacter*** | 15 (68.2%) | 6 (22.2%) | 8 (29.6%) | 6 (22.2%) | 0.0045 (0.0055) | 0.0008 (0.002) | 0.007 (0.0185) | 0.0015 (0.0042) | |
| ***Acinetobacter*** | 14 (63.6%) | 14 (51.9%) | 17 (63.0%) | 14 (51.9%) | 0.0141 (0.0234) | 0.0027 (0.0038) | 0.0034 (0.0049) | 0.0041 (0.0074) | |
| ***Finegoldia*** | 14 (63.6%) | 14 (51.9%) | 14 (51.9%) | 16 (59.3%) | 0.021 (0.0488) | 0.0216 (0.0485) | 0.0173 (0.031) | 0.0165 (0.0333) | |
| ***Actinomyces*** | 14 (63.6%) | 16 (59.3%) | 17 (63.0%) | 18 (66.7%) | 0.0166 (0.0547) | 0.0086 (0.0188) | 0.0061 (0.0088) | 0.0118 (0.0331) | |
| ***TM7 genera incertae sedis*** | 14 (63.6%) | 15 (55.6%) | 14 (51.9%) | 16 (59.3%) | 0.0026 (0.0035) | 0.0071 (0.0172) | 0.0032 (0.006) | 0.0035 (0.0056) | |
| ***Neisseria*** | 14 (63.6%) | 12 (44.4%) | 12 (44.4%) | 11 (40.7%) | 0.0055 (0.0163) | 0.0052 (0.0147) | 0.0052 (0.016) | 0.0103 (0.0466) | |
| ***Anaerococcus*** | 13 (59.1%) | 9 (33.3%) | 16 (59.3%) | 18 (66.7%) | 0.0084 (0.0196) | 0.0143 (0.0492) | 0.032 (0.0805) | 0.0138 (0.0238) | |
| ***Haemophilus*** | 13 (59.1%) | 16 (59.3%) | 15 (55.6%) | 14 (51.9%) | 0.0074 (0.0182) | 0.0141 (0.0232) | 0.0202 (0.0719) | 0.008 (0.0194) | |
| ***Porphyromonas*** | 13 (59.1%) | 10 (37.0%) | 15 (55.6%) | 12 (44.4%) | 0.0176 (0.0458) | 0.0133 (0.06) | 0.0226 (0.0731) | 0.0118 (0.0238) | |
| ***Gemella*** | 12 (54.6%) | 10 (37.0%) | 13 (48.2%) | 13 (48.2%) | 0.0029 (0.0046) | 0.0026 (0.006) | 0.0016 (0.0025) | 0.0037 (0.0071) | |
| ***Flavobacterium*** | 12 (54.6%) | 8 (29.6%) | 12 (44.4%) | 10 (37.0%) | 0.0022 (0.004) | 0.0016 (0.0041) | 0.0021 (0.0038) | 0.0077 (0.0282) | |
| ***Massilia*** | 12 (54.6%) | 4 (14.8%) | 5 (18.5%) | 12 (44.4%) | 0.0055 (0.0096) | 0.0012 (0.0032) | 0.0007 (0.0017) | 0.0027 (0.0054) | |
| ***Peptoniphilus*** | 11 (50.0%) | 11 (40.7%) | 13 (48.2%) | 15 (55.6%) | 0.0091 (0.0294) | 0.0125 (0.031) | 0.0111 (0.0191) | 0.0166 (0.0229) | |
| ***Sphingomonas CI<0.80*** | 11 (50.0%) | 7 (25.9%) | 10 (37.0%) | 13 (48.2%) | 0.0031 (0.007) | 0.0005 (0.0008) | 0.0011 (0.0023) | 0.005 (0.02) | |
| ***Microbacterium*** | 11 (50.0%) | 12 (44.4%) | 9 (33.3%) | 10 (37.0%) | 0.002 (0.0039) | 0.0012 (0.0038) | 0.0006 (0.0012) | 0.0009 (0.0015) | |
| ***Pseudonocardia*** | 11 (50.0%) | 1 (3.7%) | 7 (25.9%) | 9 (33.3%) | 0.0017 (0.0028) | 0.0002 (0.0012) | 0.0023 (0.0066) | 0.0024 (0.007) | |
| ***Veillonella*** | 10 (45.5%) | 14 (51.9%) | 17 (63.0%) | 16 (59.3%) | 0.0035 (0.0068) | 0.0307 (0.0705) | 0.0229 (0.0521) | 0.0183 (0.0383) | |
| ***Haemophilus CI<0.80*** | 10 (45.5%) | 17 (63.0%) | 18 (66.7%) | 13 (48.2%) | 0.0021 (0.0035) | 0.0094 (0.0258) | 0.0039 (0.0068) | 0.0049 (0.0108) | |
| ***Singulisphaera*** | 10 (45.5%) | 10 (37.0%) | 11 (40.7%) | 10 (37.0%) | 0.0042 (0.0091) | 0.015 (0.0404) | 0.0074 (0.016) | 0.0088 (0.0229) | |
| ***Marmoricola*** | 10 (45.5%) | 6 (22.2%) | 12 (44.4%) | 7 (25.9%) | 0.0009 (0.0016) | 0.0008 (0.0019) | 0.0022 (0.0038) | 0.0012 (0.0035) | |
| ***Cyanobacteria.GpI*** | 9 (40.9%) | 9 (33.3%) | 12 (44.4%) | 10 (37.0%) | 0.022 (0.0693) | 0.0042 (0.0094) | 0.0115 (0.0314) | 0.0079 (0.0206) | |
| ***Pasteurella CI<0.80*** | 9 (40.9%) | 12 (44.4%) | 13 (48.2%) | 8 (29.6%) | 0.0158 (0.0442) | 0.0165 (0.0415) | 0.0171 (0.0416) | 0.0308 (0.0857) | |
| ***Deinococcus*** | 9 (40.9%) | 10 (37.0%) | 8 (29.6%) | 12 (44.4%) | 0.0066 (0.0253) | 0.0009 (0.0019) | 0.0018 (0.0054) | 0.0075 (0.0235) | |
| ***Marmoricola CI<0.80*** | 9 (40.9%) | 4 (14.8%) | 9 (33.3%) | 11 (40.7%) | 0.0014 (0.0031) | 0.0018 (0.0066) | 0.0016 (0.0035) | 0.0009 (0.0016) | |
| ***Campylobacter*** | 9 (40.9%) | 10 (37.0%) | 7 (25.9%) | 11 (40.7%) | 0.0173 (0.0444) | 0.0238 (0.0705) | 0.0161 (0.0582) | 0.0145 (0.0399) | |
| ***Paracoccus*** | 8 (36.4%) | 13 (48.2%) | 16 (59.3%) | 12 (44.4%) | 0.0032 (0.0078) | 0.0197 (0.0788) | 0.01 (0.0356) | 0.0124 (0.0304) | |
| ***Corynebacteriaceae CI<0.80*** | 8 (36.4%) | 5 (18.5%) | 10 (37.0%) | 5 (18.5%) | 0.0009 (0.0018) | 0.0004 (0.0011) | 0.0006 (0.0009) | 0.0006 (0.0014) | |
| ***Bacillus CI<0.80*** | 8 (36.4%) | 4 (14.8%) | 6 (22.2%) | 7 (25.9%) | 0.0005 (0.0009) | 0.0003 (0.0005) | 0.0009 (0.0032) | 0.0005 (0.0009) | |
| ***Solirubrobacter*** | 8 (36.4%) | 9 (33.3%) | 9 (33.3%) | 10 (37.0%) | 0.0015 (0.004) | 0.0005 (0.0011) | 0.0011 (0.0028) | 0.0026 (0.0053) | |
| ***Conexibacteraceae CI<0.80*** | 8 (36.4%) | 1 (3.7%) | 6 (22.2%) | 11 (40.7%) | 0.001 (0.0025) | 0.0005 (0.002) | 0.0006 (0.0016) | 0.0015 (0.003) | |
| ***Nocardiaceae CI<0.80*** | 7 (31.8%) | 5 (18.5%) | 10 (37.0%) | 5 (18.5%) | 0.0012 (0.0034) | 0.0005 (0.0017) | 0.0012 (0.0021) | 0.0007 (0.0016) | |
| ***Dietziaceae CI<0.80*** | 7 (31.8%) | 4 (14.8%) | 11 (40.7%) | 7 (25.9%) | 0.0005 (0.0008) | 0.0003 (0.0008) | 0.0023 (0.0065) | 0.001 (0.0026) | |
| ***Sphingomonadales CI<0.80*** | 6 (27.3%) | 5 (18.5%) | 5 (18.5%) | 7 (25.9%) | 0.0027 (0.0069) | 0.0021 (0.0092) | 0.0002 (0.0005) | 0.0003 (0.0004) | |
| ***Fusobacterium*** | 6 (27.3%) | 7 (25.9%) | 9 (33.3%) | 12 (44.4%) | 0.0009 (0.002) | 0.0015 (0.0042) | 0.004 (0.0136) | 0.003 (0.005) | |
| ***Beijerinckiaceae CI<0.80*** | 6 (27.3%) | 8 (29.6%) | 10 (37.0%) | 14 (51.9%) | 0.0026 (0.0087) | 0.0017 (0.0034) | 0.0012 (0.002) | 0.0027 (0.0064) | |
| ***Pseudonocardiaceae CI<0.80*** | 6 (27.3%) | 8 (29.6%) | 7 (25.9%) | 2 (7.4%) | 0.0008 (0.0021) | 0.0008 (0.0017) | 0.0004 (0.0008) | 0.0002 (0.0008) | |
| ***Methylobacteriaceae CI<0.80*** | 6 (27.3%) | 8 (29.6%) | 3 (11.1%) | 10 (37.0%) | 0.0006 (0.0011) | 0.0007 (0.0015) | 0.0002 (0.0006) | 0.0008 (0.0013) | |
| ***Ornithinimicrobium*** | 5 (22.7%) | 6 (22.2%) | 8 (29.6%) | 9 (33.3%) | 0.0004 (0.0009) | 0.0014 (0.0038) | 0.0007 (0.0017) | 0.0007 (0.0013) | |

**Table S2.** **Results from the indicator analysis showing semen bacterial genera based on HIV infection status.**

| **Study Group** | **Semen bacteria** | **Indicator Value** | **p-value** |
| --- | --- | --- | --- |
| **HIV-uninfected** | ***Enhydrobacter*** | 0.66 | *0.001* |
|  | ***Pseudonocardia*** | 0.48 | *0.001* |
|  | ***Bifidobacterium*** | 0.36 | *0.001* |
|  | ***Geodermatophilaceae.CI<0.8*** | 0.74 | *0.006* |
|  | ***Micromonosporaceae.CI<0.8*** | 0.42 | *0.006* |
|  | ***Massilia.CI<0.8*** | 0.46 | *0.008* |
|  | ***Erwinia.CI<0.8*** | 0.34 | *0.011* |
|  | ***Leifsonia.CI<0.8*** | 0.32 | *0.011* |
|  | ***Acidovorax.CI<0.8*** | 0.23 | *0.01* |
|  | ***Friedmanniella*** | 0.45 | *0.016* |
|  | ***Nakamurellaceae.CI<0.8*** | 0.37 | *0.017* |
|  | ***Acinetobacter*** | 0.61 | *0.033* |
|  | ***Bacillus*** | 0.61 | *0.037* |
|  | ***Massilia*** | 0.45 | *0.039* |
|  | ***Hymenobacter*** | 0.39 | *0.034* |
|  | ***Rhodoferax.CI<0.8*** | 0.32 | *0.036* |
|  | ***Actinomycetales.CI<0.8*** | 0.32 | *0.029* |
|  | ***Bacteroidetes Family*** | 0.28 | *0.023* |
|  | ***Burkholderiales.CI<0.8*** | 0.27 | *0.025* |
|  | ***Variovorax.CI<0.8*** | 0.26 | *0.036* |
|  | ***Rhodobacterales.CI<0.8*** | 0.18 | *0.033* |
|  | ***Tetrasphaera.CI<0.8*** | 0.22 | *0.044* |
|  | ***Ruminococcaceae.CI<0.8*** | 0.21 | *0.047* |
| **HIV-infected, treatment-naïve** | ***Veillonella*** | 0.60 | *0.038* |
|  | ***Actinomycetospora*** | 0.34 | *0.028* |

**Table S3. Semen bacteria unique in HIV-infected men after six months of antiretroviral treatment.**

| **Study Group** | **Semen bacteria** | **Indicator Value** | **p-value** |
| --- | --- | --- | --- |
| **Six months after ART** | ***Tetrasphaera.C<0.8*** | 0.39 | *0.001* |
|  | ***Pedobacter*** | 0.38 | *0.001* |
|  | ***Curtobacterium.C<0.8*** | 0.3 | *0.004* |
|  | ***Micromonospora.C<0.8*** | 0.24 | *0.008* |
|  | ***Amaricoccus.C<0.8*** | 0.26 | *0.01* |
|  | ***Leifsonia.C<0.8*** | 0.31 | *0.023* |
|  | ***Amaricoccus*** | 0.21 | *0.025* |
|  | ***Burkholderiales.C<0.8*** | 0.18 | *0.026* |
|  | ***Clostridiales.C<0.8*** | 0.16 | *0.023* |
|  | ***Paracoccus.C<0.8*** | 0.13 | *0.032* |

**Table S4. Loadings from the principal component analysis of pro-inflammatory cytokines that correlated with semen bacterial load.**

| **Cytokine*** | **PCA1** | **PCA2** | **PCA3** | **Assignment** |  |  |
| --- | --- | --- | --- | --- | --- | --- |
| **MIG** | 0.33 | 0.10 | **0.93** | PCA 3 |  |  |
| **IL10** | 0.60 | 0.58 | 0.38 | PCA 1-3 |  |  |
| **IL17** | **0.91** | 0.24 | 0.29 | PCA 1 |  |  |
| **IL1b** | 0.16 | **0.95** | 0.05 | PCA 2 |  |  |
| **IL6** | **0.79** | 0.45 | 0.32 | PCA 1 |  |  |
| **IP10** | **0.91** | 0.03 | 0.22 | PCA 1 |  |  |
| **TNF-a** | **0.83** | 0.43 | 0.28 | PCA 1 |  |  |
| *All cytokines were log10-transformed | | | | | |  |
